# A hypoxia–glycolysis–lactate-related gene signature for prognosis prediction in hepatocellular carcinoma

**DOI:** 10.1186/s12920-024-01867-x

**Published:** 2024-04-16

**Authors:** Xiaodan Qin, Huiling Sun, Shangshang Hu, Yuqin Pan, Shukui Wang

**Affiliations:** General Clinical Research Center, Nanjing First Hospital, Nanjing Medical University, No. 68, Changle Road, 210006 Nanjing, Jiangsu China

**Keywords:** Hepatocellular carcinoma (HCC), Hypoxia, Glycolysis, Lactate, Prognosis, Immune

## Abstract

**Background:**

Liver cancer ranks sixth in incidence and third in mortality globally and hepatocellular carcinoma (HCC) accounts for 90% of it. Hypoxia, glycolysis, and lactate metabolism have been found to regulate the progression of HCC separately. However, there is a lack of studies linking the above three to predict the prognosis of HCC. The present study aimed to identify a hypoxia–glycolysis–lactate-related gene signature for assessing the prognosis of HCC.

**Methods:**

This study collected 510 hypoxia-glycolysis-lactate genes from Molecular Signatures Database (MSigDB) and then classified HCC patients from TCGA-LIHC by analyzing their hypoxia-glycolysis-lactate genes expression. Differentially expressed genes (DEGs) were screened out to construct a gene signature by LASSO-Cox analysis. Univariate and multivariate regression analyses were used to evaluate the independent prognostic value of the gene signature. Analyses of immune infiltration, somatic cell mutations, and correlation heatmap were conducted by “GSVA” R package. Single-cell analysis conducted by “SingleR”, “celldex”, “Seurat”, and “CellCha” R packages revealed how signature genes participated in hypoxia/glycolysis/lactate metabolism and PPI network identified hub genes.

**Results:**

We classified HCC patients from TCGA-LIHC into two clusters and screened out DEGs. An 18-genes prognostic signature including CDCA8, CBX2, PDE6A, MED8, DYNC1LI1, PSMD1, EIF5B, GNL2, SEPHS1, CCNJL, SOCS2, LDHA, G6PD, YBX1, RTN3, ADAMTS5, CLEC3B, and UCK2 was built to stratify the risk of HCC. The risk score of the hypoxia-glycolysis-lactate gene signature was further identified as a valuable independent factor for estimating the prognosis of HCC. Then we found that the features of clinical characteristics, immune infiltration, somatic cell mutations, and correlation analysis differed between the high-risk and low-risk groups. Furthermore, single-cell analysis indicated that the signature genes could interact with the ligand-receptors of hepatocytes/fibroblasts/plasma cells to participate in hypoxia/glycolysis/lactate metabolism and PPI network identified potential hub genes in this process: CDCA8, LDHA, YBX1.

**Conclusion:**

The hypoxia–glycolysis–lactate-related gene signature we built could provide prognostic value for HCC and suggest several hub genes for future HCC studies.

**Supplementary Information:**

The online version contains supplementary material available at 10.1186/s12920-024-01867-x.

## Introduction

Liver cancer ranks sixth in incidence and third in mortality globally [1]. Hepatocellular carcinoma (HCC) accounts for approximately 90% of all liver cancer cases [2]. While significant advancements have been made in therapeutic approaches for HCC, such as surgical resection, radiofrequency ablation, and orthotopic liver transplantation [3], the prognosis for HCC patients remains unsatisfactory [4]. Heterogeneity within HCC presents challenges in both diagnosis and treatment, making accurate prognosis estimation and personalized treatment for patients a complex task [5, 6]. Therefore, seeking a more reliable prognostic prediction model for HCC is significant.

The microenvironment of solid tumors is often characterized by hypoxia, resulting from an aberrant vascular system that fails to deliver sufficient oxygen to meet the rapid proliferation demands of tumor cells [7]. The liver, being highly susceptible to hypoxia, exhibits a microenvironment in HCC that is both hypoxic and nutrition deficient [8]. Tumors adapt to hypoxia by activating hypoxia-inducible factors (HIFs), which play a crucial role in facilitating a shift towards anaerobic energy production [9]. The acceleration of cellular glycolysis by hypoxia-inducible factor-1α (HIF-1α) has been documented as a means to sustain energy supply, impacting both cancer cells and healthy cells [10]. Tumor-associated aberrant glycolysis can result in elevated lactate production, thereby influencing the pH of the tumor microenvironment and affecting both cancer cells and immune cells [11]. Previous research has indicated that the knockout of aldolase A (ALDOA), a crucial enzyme in glycolysis and gluconeogenesis, can deplete lactate levels in HCC and impede tumor growth [12].

Recent studies have confirmed that hypoxia, glycolysis, and lactate metabolism are crucial for HCC development. Previous prognostic models of HCC were constructed based on the individual effects of hypoxia or glycolysis or lactate metabolism separately. For example, Hu et al. found hypoxia/immune-associated prognosis signature can stratify the risk of HCC [13]. Chen et al. found glycolysis/gluconeogenesis-related genes were associated with patient prognosis, immune microenvironment, and immunotherapy response in HCC [14]. Cheng et al. found the lactylation-related gene signature could serve as a biomarker for the clinical treatment of HCC [15]. However, upon the integration of previous studies, we conjectured that hypoxia, glycolysis, and lactate metabolism do not function independently but interact to regulate HCC. Therefore, we comprehensively discussed their combined prognostic value of HCC for the first time. Subsequently, we developed a hypoxia-glycolysis-lactate-related gene signature and systematically examined its implications through survival analysis, clinical characteristics, immune infiltration, somatic cell mutations, correlation analysis, and single-cell analysis, which filled this particular gap in the prognostic models for HCC.

## Materials and methods

### Data collection

We downloaded the complete RNA expression matrix along with the relevant clinical data for 377 HCC patients from the TCGA database (http://portal.gdc.cancer.gov/repository). Additionally, we obtained the RNA expression matrix and associated clinical data of 247 HCC patients from the GEO database (https://www.ncbi.nlm.nih.gov/geo/). These raw data were collated and normalized through the “limma” R package [16]. Detailed clinicopathological characteristics of HCC patients in TCGA-LIHC and GSE14520 were provided in Table S1 of the supplementary materials.

### Genes collection

A total of 200 hypoxia genes, 200 glycolysis genes, and 303 lactate genes were acquired via the human gene sets in the Molecular Signatures Database (MSigDB) [17] (http://www.gsea-msigdb.org/gsea/msigdb). After excluding the same genes among the three gene sets, we collected 510 related genes. These 510 related genes were further utilized to classify HCC patients from TCGA into two subtypes, among which we screened out DEGs.

### Consistency clustering analysis and clinical traits analysis between different subtypes

The “Consensus Cluster Plus” R package [18] was used to classify the HCC patients in the TCGA into two major subtypes. The heatmap was conducted to test whether there was a difference between the two subtypes regarding specific clinical characteristics. We also conducted a Kaplan-Meier survival analysis to compare the survival probability of the two subtypes. Moreover, the heatmap of two subtypes from KEGG gene sets and hallmark gene sets of the MSigDB was conducted to explore the differential gene sets between the two subtypes.

### DEGs among two HCC subtypes and functional enrichment analysis

We utilized the R package “ggplot2” [19] to identify the DEGs between the two subtypes of HCC. Genes with|log2 fold change (FC)| > 1 and false discovery rate (FDR) < 0.05 were defined as differentially expressed. Afterwards, we visualized the results through a volcano plot. Enrichment analyses of Gene Ontology (GO) and Kyoto Encyclopedia of Genes and Genomes pathway (KEGG) were conducted to explore various functional pathways associated with DEGs. GO enrichment analysis included the biological process (BP), molecular function (MF), and cellular component (CC).

### Construction and validation of a hypoxia-glycolysis-lactate related gene signature

We defined 370 HCC patients in TCGA-LIHC as the training cohort and 220 HCC patients in GSE14520 as the test cohort. We performed univariate regression analysis to identify potential prognostic genes the criteria of p value < 0.001 [20]. The least absolute shrinkage and selection operator (LASSO) is a regularization method for linear regression problems that can reduce model complexity, avoid overfitting, and select important eigenvariables. The principle of LASSO is to minimize the sum of residual squares, resulting in certain regression coefficients equal to 0, thus obtaining an optimal model. Therefore, we utilized the LASSO Cox regression analysis [21] along with “glmnet” R package [22] to identify genes with strong correlation. Finally, a related gene signature was constructed. The risk score was calculated as the sum of multiplication with gene expression and correlation gene coefficients. Subsequently, HCC patients in both cohorts were categorized into high-risk and low-risk groups based on the median risk score value. The survival state and survival probability of HCC patients in both the training cohort and test cohort were explored through the “pheatmap” R package.

### Construction and evaluation of the nomogram

Using the “rms” R package, we constructed a nomogram that included the risk score and clinical features as prognostic factors. Based on the overall score, we could forecast the 1-, 3-, and 5-year survival probability of HCC patients. Calibration curves and ROC curves were plotted to evaluate the nomogram. We also conducted univariate and multivariate Cox regression analyses to assess the potential of risk scores as an independent prognostic factor for predicting HCC patient prognosis.

### Analysis of immune cell infiltration, immune function, somatic mutation, and correlation analysis

The “GSVA” R package was used to perform single-sample gene set enrichment (ssGSEA) analysis to explore the immune cell infiltration and immune function of each sample [23]. Meanwhile, the TIDE score [24] and ROC curve were used to investigate the difference in immunotherapy sensitivity between high-risk and low-risk groups. Moreover, the waterfall diagram of somatic mutation distribution was drawn to identify genes with the highest frequency of mutations. The “GSVA” R package was also used to conduct the heatmap of correlation analysis between risk score and hallmark pathways.

### Analysis of single‑cell RNA sequencing (scRNA‑seq)

We downloaded single-cell RNA sequencing (scRNA-Seq) data from GSE18993 through the GEO database. Firstly, genes with significant variance were screened out via the “Seurat” R package for subsequent analysis. Dimensional reduction of these genes was then conducted through PCA. Finally, we used Uniform Manifold Approximation and Projection (UMAP) to identify different cell clusters and annotated each cell cluster through “SingleR” and “celldex” R packages.

### Single-cell gene set scoring, cell-cell communication analysis, and protein-protein Interaction (PPI) network construction

Single-cell sequencing data were scored using the “AddModuleScore” function of the “Seurat” R package. The “HALLMARK” and “KEGG” gene sets were obtained from the Molecular Signatures Database (https://www.gsea-msigdb.org/gsea/msigdb). Cell-cell communication analysis was performed using the R software “CellChat” package [25]. Protein-Protein Interaction (PPI) network was constructed based on the STRING (https://cn.string-db.org/) database. Set condition: minimum required interaction score is 0.4.

## Results

### Hypoxia-high, glycolysis-high, and lactate-high HCC patients had a lower survival probability

**Fig. 1:**
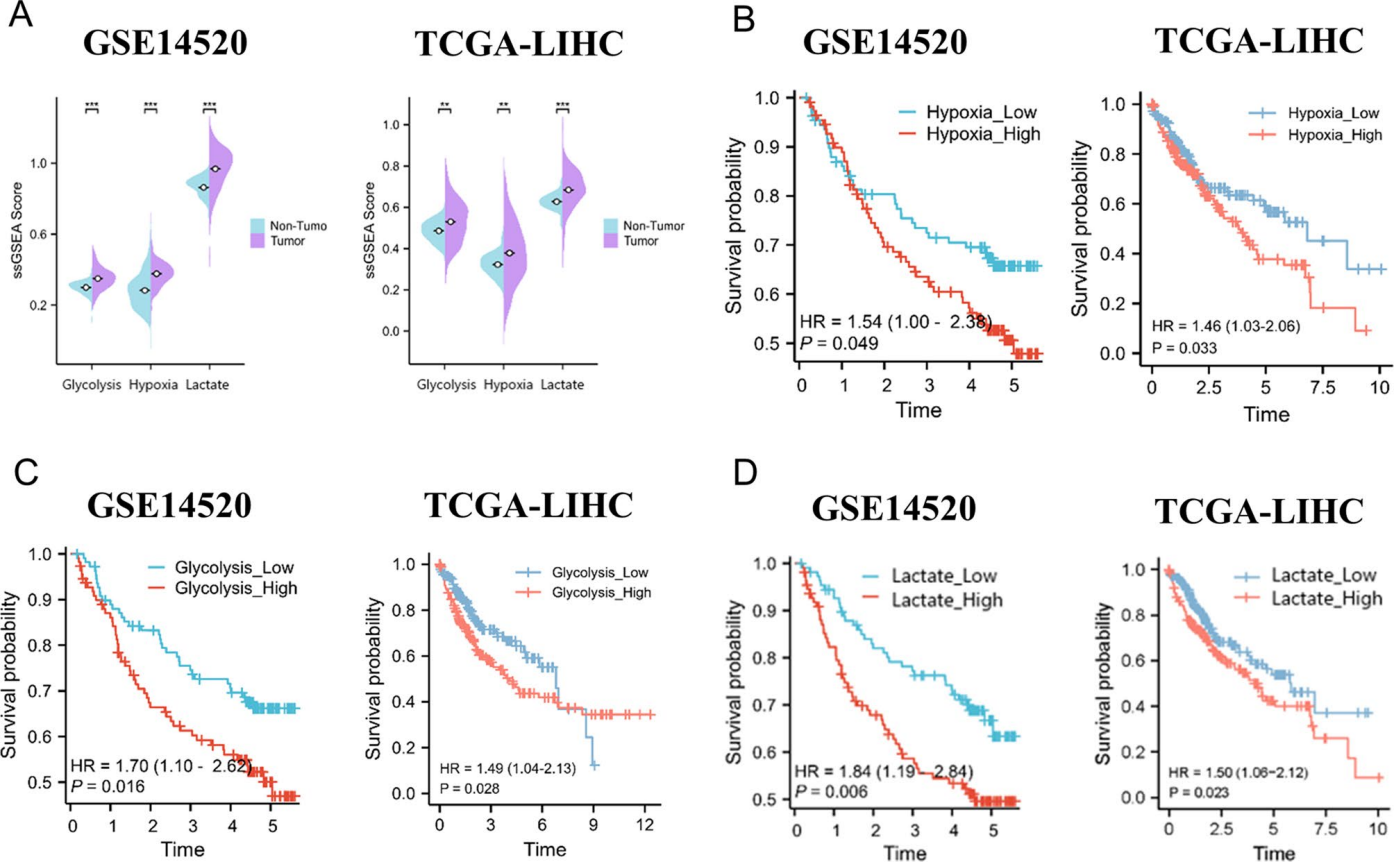
ssGSEA scores and Kaplan-Meier survival analysis of glycolysis, hypoxia, and lactate genes in the GEO and TCGA cohorts. (**A**) ssGSEA scores of glycolysis, hypoxia, and lactate genes in GSE14520 (left) and TCGA–LIHC (right). (**B**– **D**) Kaplan-Meier survival analysis of glycolysis, hypoxia, and lactate genes in the GSE14520 (left) and TCGA-LIHC (right)

Through ssGSEA score, we found that hypoxia, glycolysis, and lactate gene sets were more likely enriched in HCC samples than non-tumor samples in both TCGA-LIHC and GSE14520 (Fig. 1A). The HCC patients from TCGA and GEO databases were stratified into two groups based on the expression of hypoxia, glycolysis, and lactate genes respectively. Through survival analysis, it was observed that patients of hypoxia-high, glycolysis-high, and lactate-high groups had a reduced survival probability than those in the hypoxia-low (Fig. 1B), glycolysis-low (Fig. 1C) and lactate-low (Fig. 1D) groups in both TCGA and GEO databases.

### Division subtypes, clinical features analysis, and survival analysis based on consensus cluster analysis

**Fig. 2:**
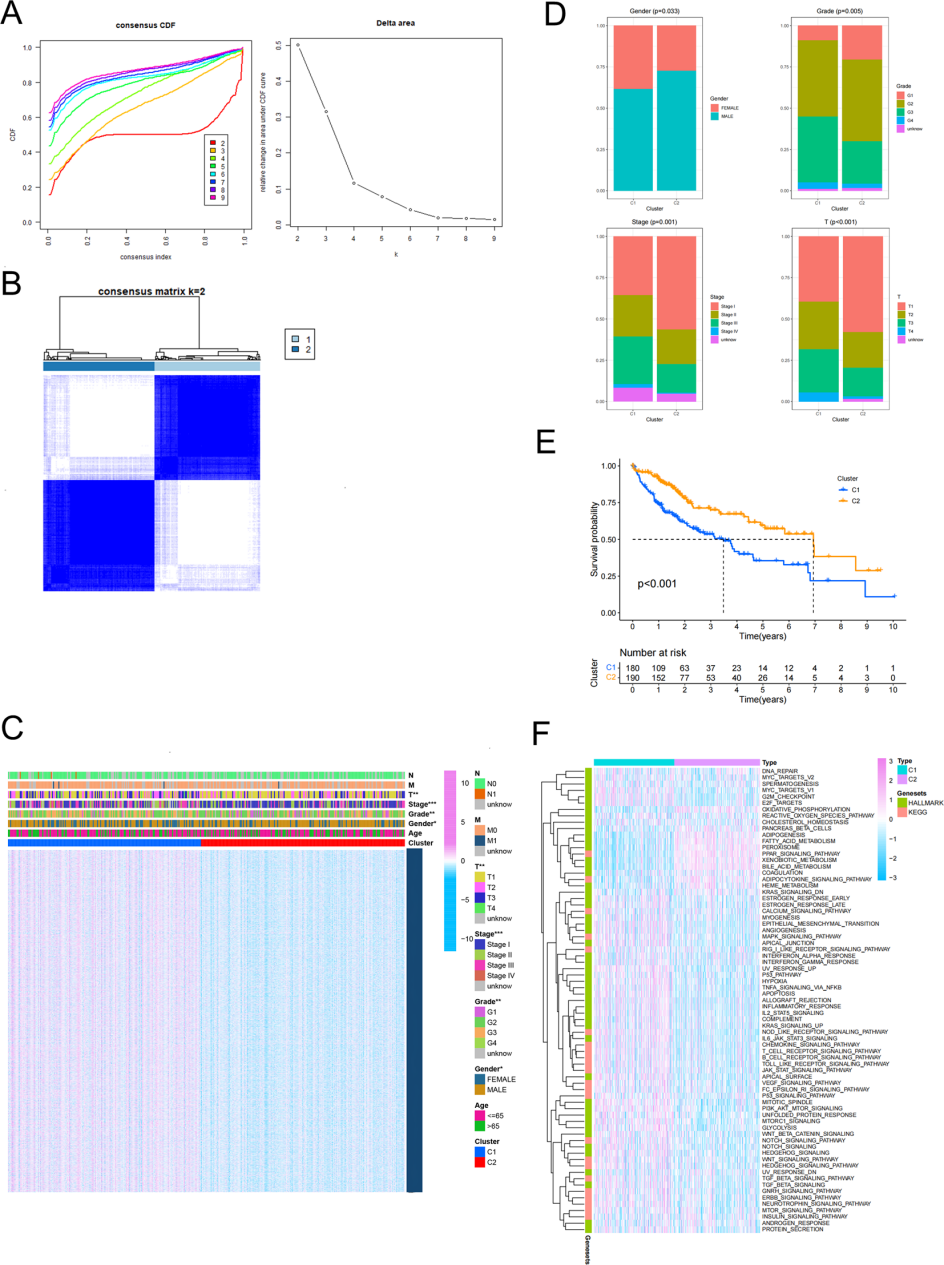
Classification of HCC subtypes and related clinical features analysis. (**A**) The CDF distribution diagram shows value of CDF under different k value, the red line with k = 2 is most smoothest (Left); the relative change in area under CDF curve is shown on the right and the largest difference is between k = 2 and k = 3. (**B**) The diagram illustrating the consistency matrix when k = 2. (**C**) Heatmap of clinical traits (TNM, stage, grade, gender, age) of two subtypes (*p < 0.05, **p < 0.01, ***p < 0.001). (**D**) Comparison of the proportion between two subtypes regarding gender, grade, stage, and T staging. (**E**) Kaplan-Meier survival analysis of two subtypes. (**F**) Heatmap of two subtypes from KEGG gene sets and hallmark gene sets of the MsigD

To identify potential prognostic genes, we performed univariate Cox regression analysis among a total of 510 hypoxia, glycolysis, and lactate-related genes. Consensus clustering analysis was subsequently performed to identify HCC subtypes. The consensus CDF plot illustrated the distribution under various numbers of clusters, suggesting a clear discrimination when the number of clusters (K) was set to 2 (Fig. 2A). Accordingly, we divided all HCC patients into two main subtypes (cluster 1 and cluster 2) in TCGA (Fig. 2B). The heatmap of clinical features between the two subtypes showed that the characteristics of the two clusters differed in gender, grade, and stage (Fig. 2C). Comparing two clusters of HCC patients, it was observed that Cluster 1 had a significantly higher number of female patients than Cluster 2. Furthermore, it was found that the proportion of HCC patients in either the middle or end stage was greater in Cluster 1 than in Cluster 2 (Fig. 2D). These findings may have important implications for classifying HCC patients. Based on the Kaplan-Meier analysis, it was observed that the survival probability of Cluster 1 (*n* = 180) was comparatively lower than that of Cluster 2 (*n* = 190) (Fig. 2E). Meanwhile, we conducted the heatmap of two subtypes from KEGG gene sets and hallmark gene sets of the MSigDB (Fig. 2F). It can be found that the process of hypoxia and glycolysis were more likely to be activated in Cluster 1 than in Cluster 2.

### Construction of a prognostic gene signature for HCC patients in the TCGA cohort

**Fig. 3:**
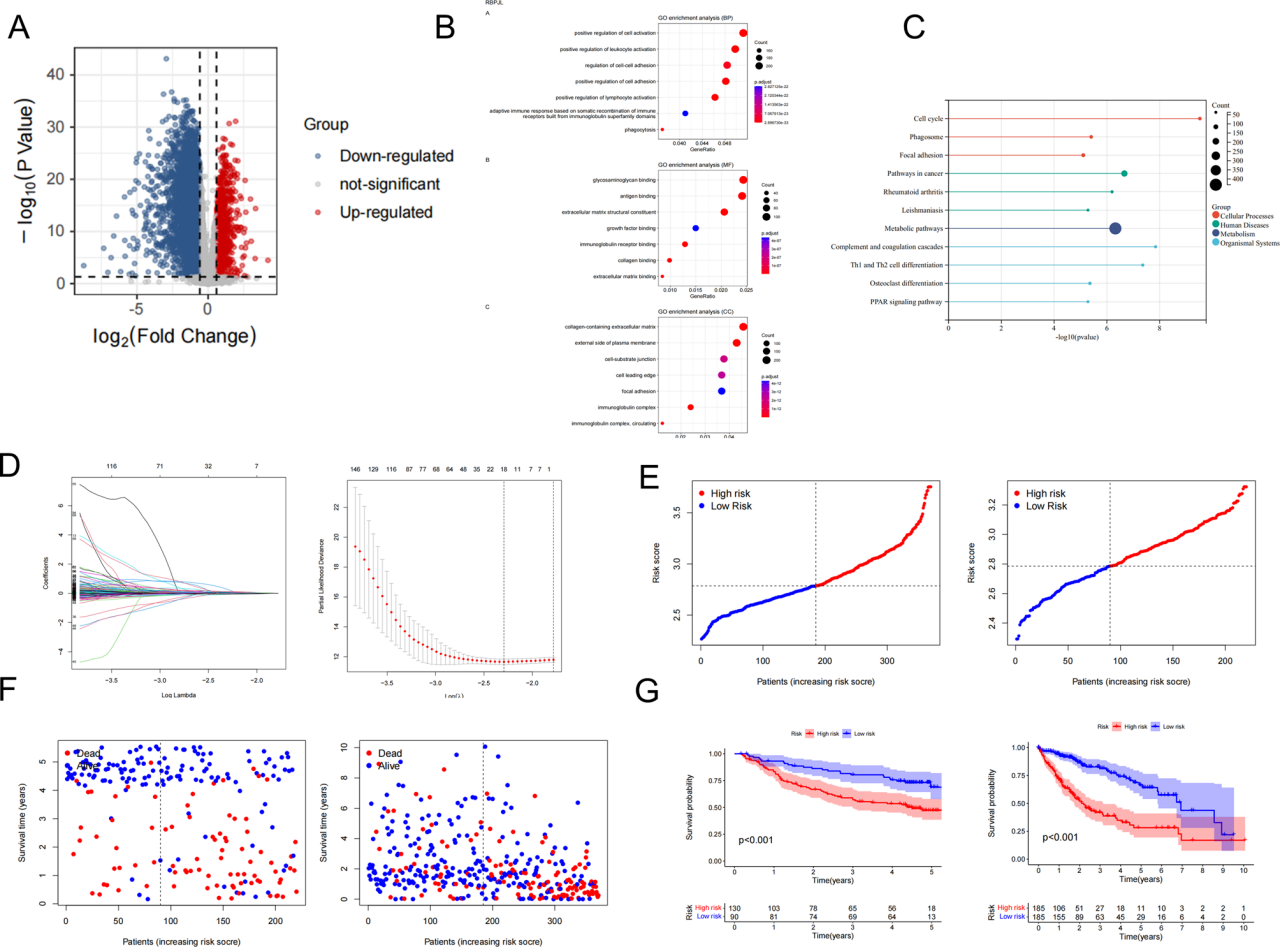
Functional enrichment analysis and prognostic gene signature construction. (**A**) A volcano plot depicting genes related to hypoxia, glycolysis, and lactate that were differentially expressed. Downregulated genes are represented in blue, upregulated genes in red, and non-differentially expressed genes in gray. (**B**) GO enrichment analysis of DEGs; BP, MF, CC are shown from top to bottom. (**C**) KEGG enrichment analysis of DEGs based on four groups: cellular processes, human diseases, metabolism, and organsimal systems. (**D**) Regression coefficient path plot (left) and cross-validation curve (right) of LASSO Cox regression analysis. (**E**– **G**) Risk curve, survival status, and Kaplan-Meier survival analysis of HCC patients under different risk groups in the training cohort (left) and test cohort (right) (***p < 0.001)

A total of thousands of DEGs between the two subtypes were identified through the “ggplot2” R package. The volcano map revealed that 716 genes were upregulated, while 4437 genes were downregulated (Fig. 3A). Further analysis was performed using GO and KEGG enrichment analyses to determine the biological processes involved in the DEGs. The analysis of GO revealed that the DEGs were mainly involved in cell activation as regards the biological process. As for the molecular function of DEGs, it was primarily enriched in glycosaminoglycan binding. The cellular component of DEGs was mainly enriched in an extracellular matrix that contained collagen (Fig. 3B). Following the KEGG analysis, it was observed that the DEGs were significantly enriched in metabolic pathways (Fig. 3C). In the training cohort samples, the univariate regression analysis was performed to identify 3664 potential prognostic genes. The identified genes were further analyzed using LASSO COX regression analysis. Through the optimal value of λ, a prognostic signature consisting of 18 genes was established. This signature included cell division cycle associated 8 (CDCA8), Chromobox 2 (CBX2), Phosphodiesterase 6 A (PDE6A), mediator complex subunit 8 (MED8), Dynein Cytoplasmic 1 Light Intermediate Chain 1 (DYNC1LI1), proteasome 26 S subunit, non-ATPase 1 (PSMD1), eukaryotic translation initiation factor 5B (EIF5B), G Protein Nucleolar 2 (GNL2), selenophosphate synthetase 1(SEPHS1), cyclin J like (CCNJL), suppressor of cytokine signaling 2 (SOCS2), lactate dehydrogenase A (LDHA), glucose-6-phosphate dehydrogenase (G6PD), Y-box binding protein 1 (YBX1), reticulon 3 (RTN3), ADAM metallopeptidase with thrombospondin type 1 motif 5 (ADAMTS5), C-type lectin domain family three member B (CLEC3B), and uridine-cytidine kinase 2 (UCK2) (Fig. 3D). The following formula calculated the risk score: risk score = [CDCA8 expression* (0.0103)] + [CBX2 expression* (0.1041)] + [PDE6A expression* (0.2492)]+ [MED8 expression* (0.0031)] + [DYNC1LI1 expression* (0.0344)]+ [PSMD1 expression* (0.0202)]+ [EIF5B expression* (0.1233)]+ [GNL2 expression* (0.0137)]+ [SEPHS1 expression* (0.0140)]+ [CCNJL expression* (0.0730)]+ [SOCS2 expression* (-0.0736)]+ [LDHA expression* (0.0510)]+ [G6PD expression* (0.0047)]+ [YBX1 expression* (0.0832)]+ [RTN3 expression* (0.0491)]+ [ADAMTS5 expression* (0.1021)]+ [CLEC3B expression* (-0.0643)]+ [UCK2 expression* (0.0200)]. All samples from the train and test cohorts were classified as high- or low-risk groups based on their median risk score value. The risk curves (Fig. 3E) and survival status plots (Fig. 3F) revealed that in both the training and test cohorts, patients at high risk had significantly reduced survival time compared to those at low risk. The survival probability of patients in the high-risk group was lower than that of patients in the low-risk group, as shown by the Kaplan-Meier analysis, which was observed in both the training and test cohorts (Fig. 3G, *p* < 0.001).

### Independent prognostic value of the 18-gene signature

**Fig. 4:**
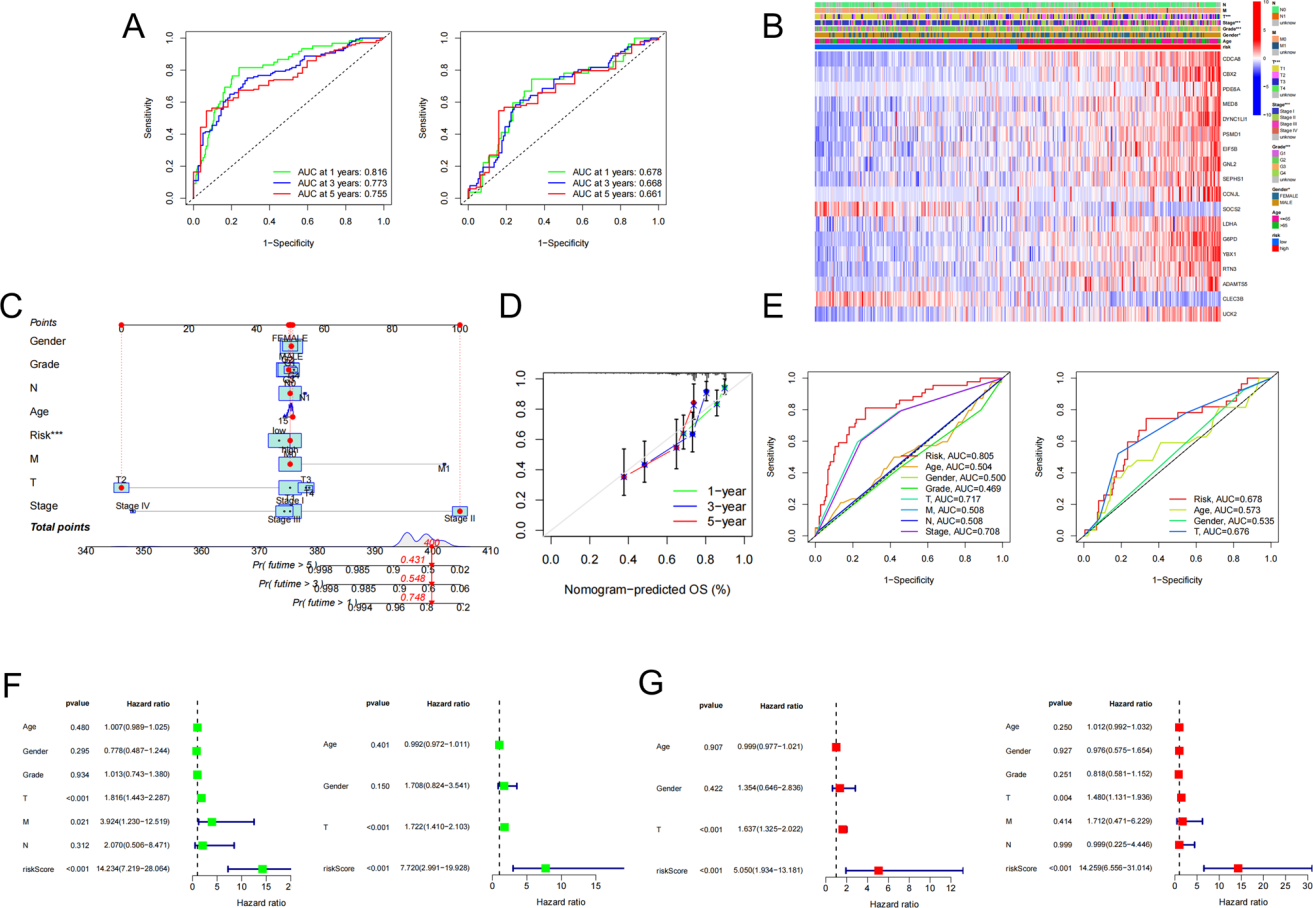
Construction of a nomogram and independent prognostic analysis of risk score. (**A**) ROC curves for 1-, 3-, and 5-year in the training cohort (left) and test cohort (right). (**B**) Heatmap of the correlation between clinical features (TNM, stage, grade, gender, age) and risk score (*p < 0.05, **p < 0.01, ***p < 0.001). (**C**) Nomogram comprised of risk score and clinical traits (TNM, stage, grade, gender, age). (**D**) Calibration plots of nomogram. (**E**) ROC curves based on clinical traits and risk score in the training cohort (left) and test cohort (right). (**F**) Univariate analysis of risk score as an independent prognostic factor in patients with HCC in the training cohort (left) and test cohort (right). (**G**) Multivariate analysis of risk score as an independent prognostic factor for HCC patients in the training cohort (left) and test cohort (right)

ROC curves were used to evaluate the accuracy of the 18-gene signature in predicting the survival rates at 1-year, 3-year, and 5-year in both the training and test cohorts, as shown in Fig. 4A. The results indicated that the AUC was greater than 0.7 for all time points, indicating satisfactory accuracy. We also explored the correlation between clinical characteristics and risk scores. The results showed that patients at advanced stages of HCC had higher risk scores (Fig. 4B). Using the “rms” R package, we developed a nomogram to predict the survival probability of HCC patients at 1-year, 3-year, and 5-year based on available clinical data and the risk score (Fig. 4C). The calibration plots (Fig. 4D) and ROC curves for both the training and test cohorts (Fig. 4E) demonstrated the accuracy of the nomogram in predicting the survival of HCC patients. We performed univariate and multivariate Cox regression analyses based on risk score and clinical traits for both the training and test cohorts to confirm that the risk score of the 18-gene signature was an independent prognostic factor for HCC patients. The risk score was found to be significantly associated with overall survival (OS) in both cohorts through univariate Cox analysis (training cohort: HR = 14.234, 95%CI = 7.219–28.064, *P* < 0.001; test cohort: HR = 7.720, 95%CI = 2.991–19.928, *P* < 0.001; Fig. 4(F)). Moreover, multivariate Cox analysis indicated that the risk score was an independent predictor for OS (training cohort: HR = 14.259, 95%CI = 6.556–31.014, *P* < 0.001; GEO cohort: HR = 5.050, 95%CI = 1.934–13.181, *P* < 0.001; Fig. 4(G)).

### Immune landscape, state of somatic mutation, and correlation analysis based on risk score

**Fig. 5:**
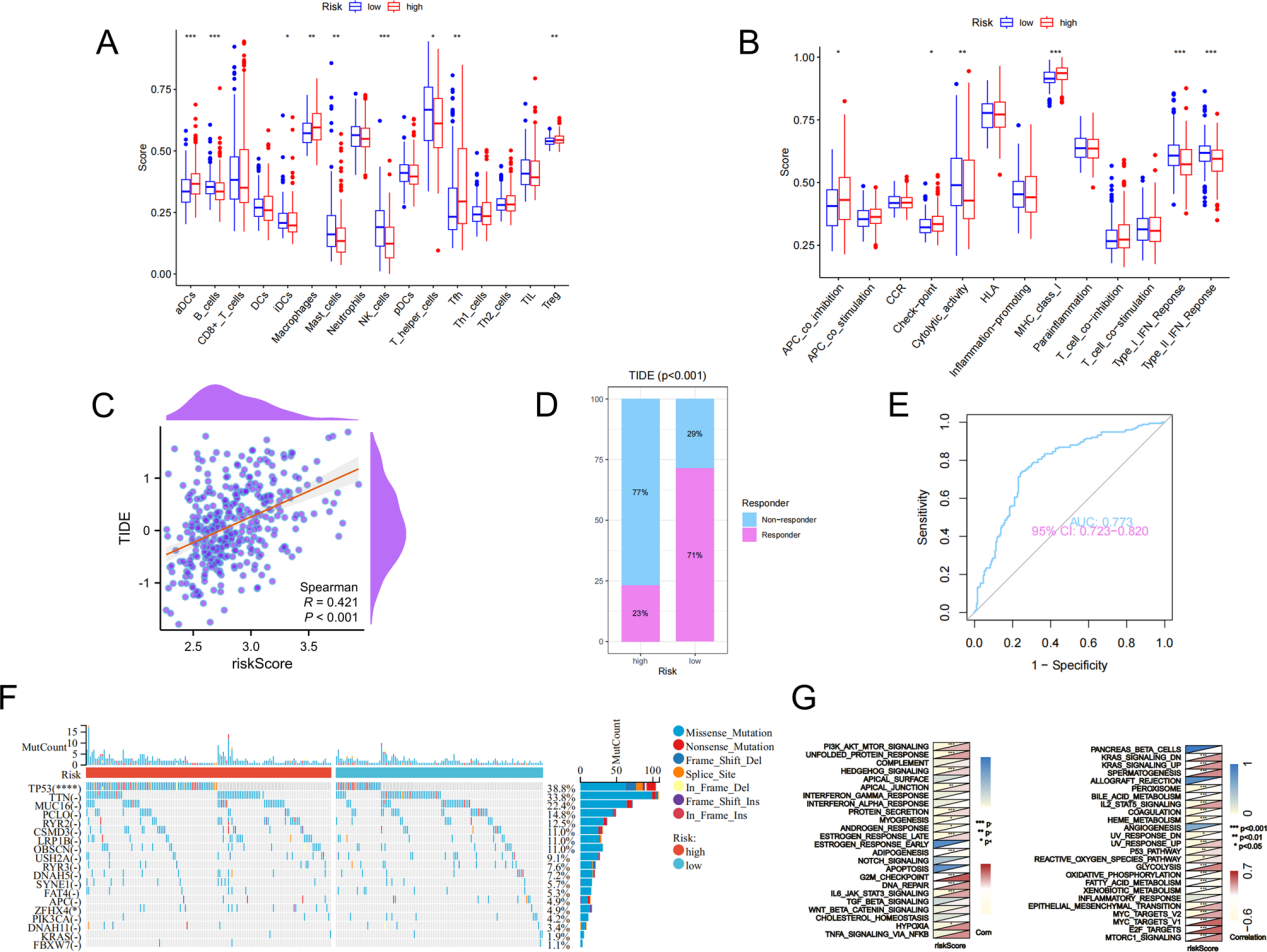
Immune cell infiltration, immune function, TIDE score, somatic mutations, and correlation analysis based on risk score. (**A**) Analysis of immune cells infiltration under different risk groups. (**B**) Analysis of immune function under different risk groups. (**C**) A graph showing the correlation between TIDE score and risk score. (**D**) Comparison of the immunotherapy response proportion between different risk groups. (**E**) ROC curves of immunotherapy response of patients with HCC. (**F**) Waterfall diagram of genes with most frequent somatic mutations under different risk groups. (**G**) Heatmap of correlation analysis between risk score and hallmark pathways. ***p < 0.001; **p < 0.01; *p < 0.05

The immune cell infiltration analysis revealed an upregulation of activated dendritic cells, macrophages, and regulatory T cells, along with a downregulation of B cells, interdigitating cells, mast cells, NK cells, and T helper cells in the high-risk group (Fig. 5A). Enrichment analysis of immune function indicated that the APC co-inhibition, checkpoint, and MHC-class-I were significantly enriched in the high-risk group. In contrast, the low-risk group exhibited a significant enrichment in cytolytic activity, Type-I-IFN-Response, and Type-II-IFN-Response (Fig. 5B). We investigated the correlation between the TIDE and risk scores (Fig. 5C) and compared the response proportion between different risk groups (Fig. 5D). Patients in the high-risk group demonstrated a higher TIDE score, suggesting a greater probability of immune evasion and lower chances of benefiting from immunotherapy. The ROC curve confirmed the accuracy of the above results, with an AUC of 0.773 (Fig. 5E). The waterfall diagram of somatic mutation distribution was drawn to explore genes with the highest mutation frequency in the two risk groups (Fig. 5F). Results showed a significantly higher frequency of TP53 and ZFHX4 mutations in the high-risk group compared to the low-risk group. The heatmap of correlation analysis via GSVA showed that the high-risk score correlated more with specific hallmark pathways such as PI3K-AKT-MTOR signaling, glycolysis, DNA repair, and so on (Fig. 5G).

### Single‑cell RNA sequencing (scRNA‑seq) analysis of HCC patients from GSE18993

**Fig. 6:**
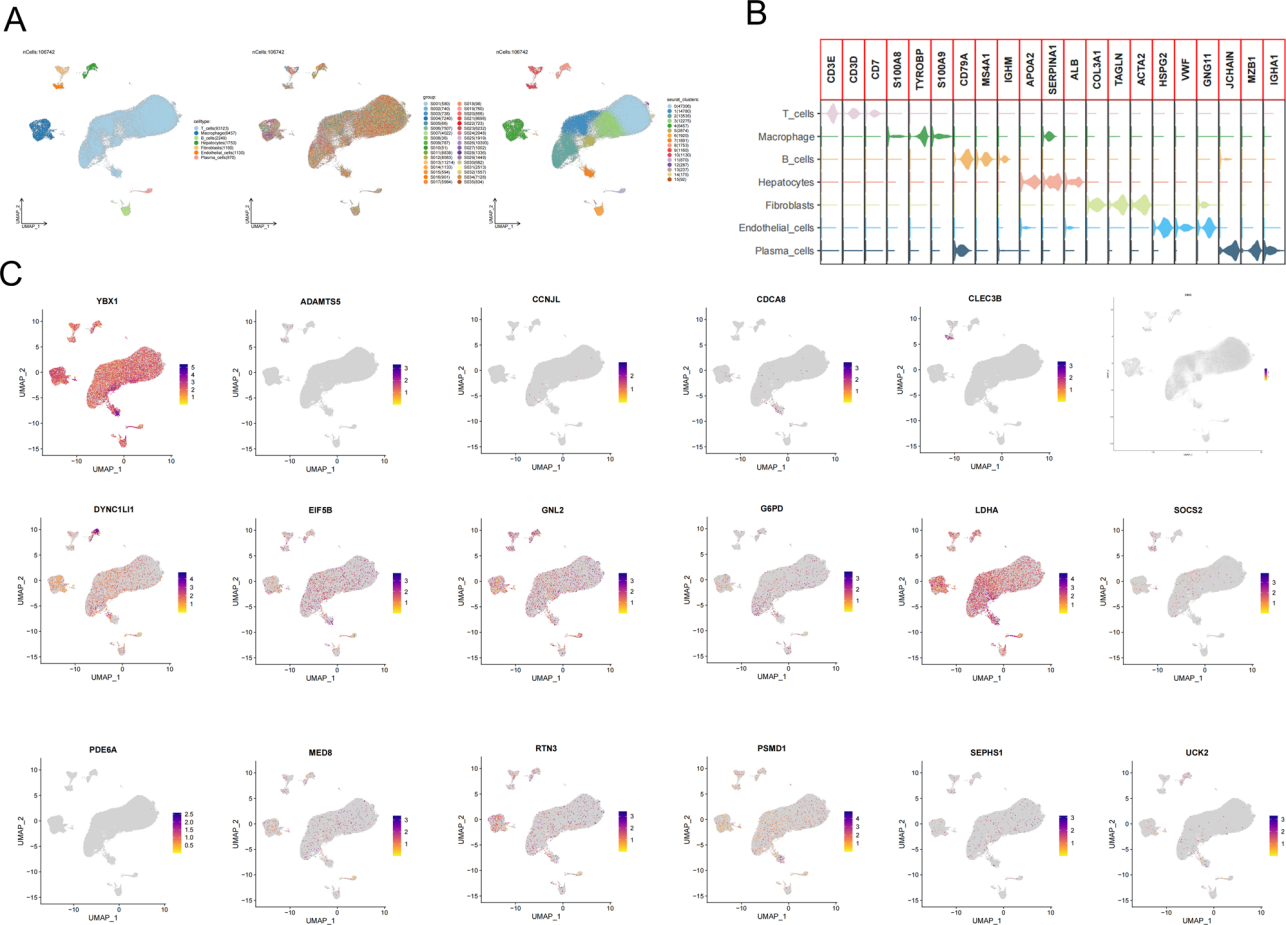
Overview of single-cell analysis from GSE18993. (**A**) UMAPs of the 7 cell types, 35 groups, and 15 clusters. (**B**) Expression of cell surface and intracellular markers in each cell type. (**C**) Feature plots showing the signature gene expression across the 7 cell types (T cells, Macrophages, B cells, Hepatocytes, Fibroblasts, Endothelial cells, and Plasma cells)

A total of 106,742 single cells from GSE18993 were successfully classified into seven cell types (T cells, Macrophages, B cells, Hepatocytes, Fibroblasts, Endothelial cells, and Plasma cells), 35 groups, and 15 clusters by UMAP algorithm (Fig. 6A). Markers of T cells, Macrophages, B cells, Hepatocytes, Fibroblasts, Endothelial cells, and Plasma cells were plotted (Fig. 6B). The expression of 18 signature genes across seven cell types was shown in Fig. 6C.

### Correlation between signature genes and hypoxia/glycolysis/lactate

**Fig. 7:**
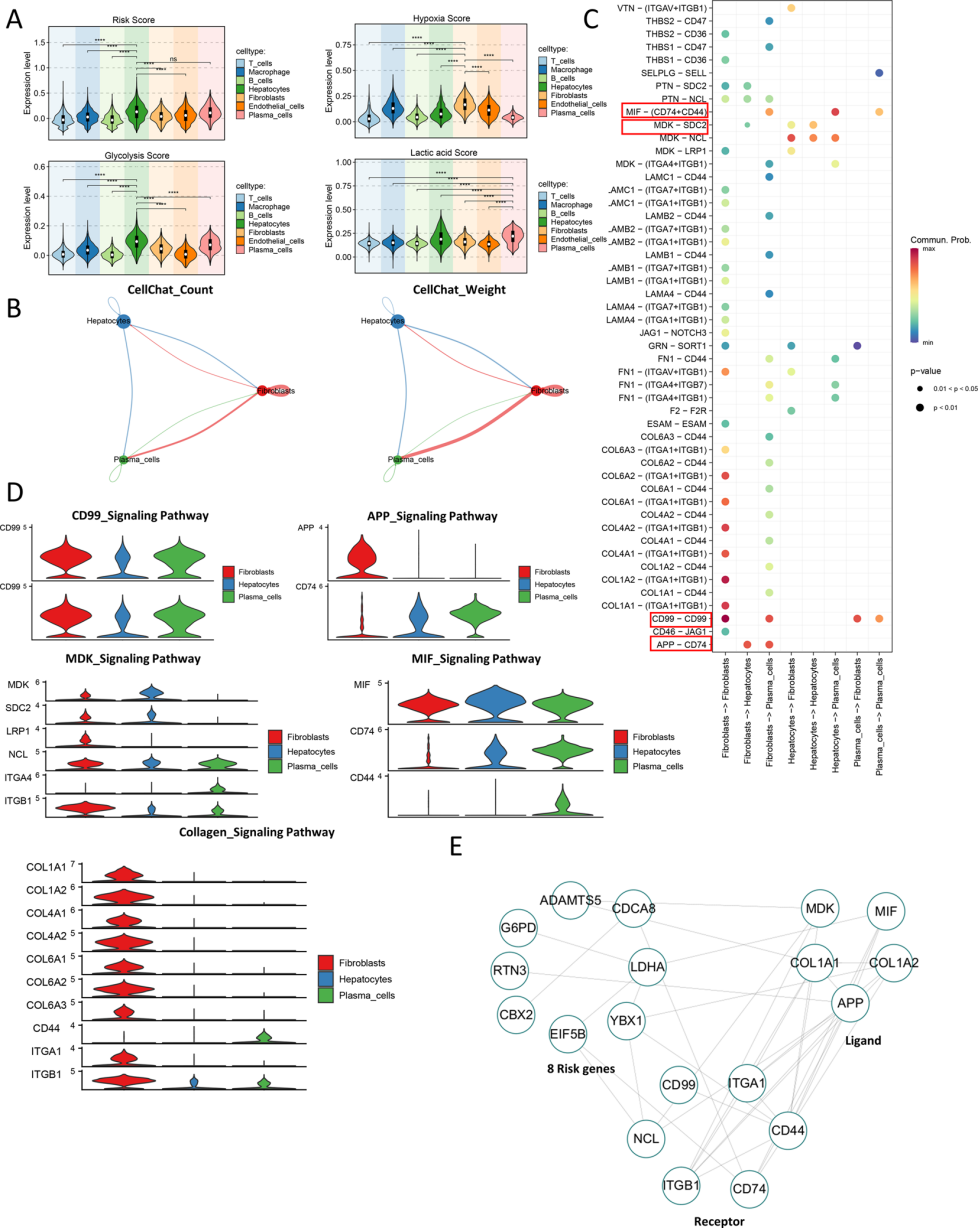
Correlation between signature genes and hypoxia/glycolysis/lactate (**A**) Scores and differential analysis of signature gene set/hypoxia gene set/glycolysis gene set/lactate gene set. (**B**) CellChat-count and the CellChat-weight of hepatocytes, fibroblasts, and plasma cells. (**C**) Bubble plot of ligand-receptor interactions with hepatocytes, fibroblasts, and plasma cells. (**D**) Expression of five pathways (APP-CD4, CD99-CD99, MDC-NCL, several Collagen receptors and ligands, and MIF-(CD74 + CD44)). (**E**) PPI network of risk genes

We further explored the correlation between signature genes and hypoxia/glycolysis/lactate at the single-cell level. Firstly, we scored the signature gene set/hypoxia gene set/glycolysis gene set/lactate gene set, and we found that the risk score and glycolysis score were the highest expressed in hepatocytes, hypoxia score was the highest expressed in fibroblasts, and the highest lactate metabolism score was the highest expressed in plasma cells (Fig. 7A). We hypothesized that hepatocytes/fibroblasts/plasma cells may play an important role in the association of signature genes and hypoxia/glycolysis/lactate. Then we calculated the CellChat-count and the CellChat-weight of hepatocytes, fibroblasts, and plasma cells. We found that there was a crosstalk between the three cell types (Fig. 7B). We predicted the ligand-receptor interactions of hepatocytes, fibroblasts, and plasma cells, with five pathways (APP-CD4, CD99-CD99, MDC-NCL, several Collagen ligand-receptors, and MIF-(CD74 + CD44)) having strong interactions between the above three cell types (Fig. 7C). The expression of these five pathways are shown in Fig. 7D. Based on the PPI network, we found that 8 risk genes (ADAMTS5, CDCA8, LDHA, YBX1, EIF5B, CBX2, RTN3, and GP6D) interacted with the ligand-receptors of hepatocytes, fibroblasts, and plasma cells (Fig. 7E). Therefore, the above 8 risk genes were involved in the process of hypoxia/glycolysis/lactate metabolism through this PPI network.

## Discussion

HCC is the most common type of liver cancer and a leading cause of death worldwide [26]. The high molecular heterogeneity of HCC has complicated the understanding of hepatocarcinogenesis and limited the clinical efficacy [27, 28]. HCC is widely reported to be metabolically heterogeneous, and the intervention of metabolic disorders has been used for HCC treatment [29]. According to metabolomics, HCC can be divided into four molecular subtypes, each with different metabolic preferences [30]. Therefore, we tended to explore a metabolic prognostic signature to predict the prognosis of HCC patients.

Hypoxia can enhance the glycolytic metabolism of cancer cells, aiding in energy generation and facilitating tumor metastasis [9]. Targeting glycolytic enzymes is a potential approach to treating HCC, and several drugs have been under investigation [31, 32]. Meanwhile, it is reported that lactate, produced by anaerobic glycolysis, could enhance ferroptosis resistance in HCC cells and contribute to tumor growth [33]. In the present study, we collected hypoxia, glycolysis, and lactate-related genes from the MSigDB database and investigated the correlation between those genes and HCC. Through consensus cluster analysis, we divided HCC patients into two subtypes and found the difference in clinical traits between the two subtypes. GO and KEGG analysis of DEGs between the two subtypes showed that DEGs were mainly enriched in cell activation, glycosaminoglycan binding, collagen-containing extracellular matrix, and metabolic pathways. The extracellular matrix (ECM) is a dense network of proteins and carbohydrates around cells, and glycosaminoglycans are one of the main components of it. Researchers at UCLA have found that ECM could regulate the movement of breast cancer cells in the body by regulating glucose consumption [34], providing insights into the potential role of ECM in the regulation of HCC.

We furthermore screened out candidate prognostic genes among DEGs and constructed an 18-gene prediction model. Among the eighteen genes, CDCA8, CBX2, PDE6A, MED8, DYNC1LI1, PSMD1, EIF5B, GNL2, SEPHS1, CCNJL, LDHA, G6PD, YBX1, RTN3, ADAMTS5, UCK2 were upregulated and SOCS2, CLEC3B were downregulated in high-risk score group. Research has shown that SOCS2 and CLEC3B were tumor suppressor genes [35, 36], which may potentially explain the above trend. Notably, all these signature genes have been reported in HCC, although investigations into specific mechanism in the disease remained limited. For instance, CDCA8 has been associated with promoting cancer progression by binding with NF-YA and has been considered a potential therapeutic target for HCC patients [37]. Increased SEPHS1 expression has been linked to poor prognosis in HCC patients [38]. The expression of LDHA was regulated by MYC through microRNA-122-5p to potentiate glycolysis in HCC [39]. YB1 could regulate miR-205/200b-ZEB1 axis to promote the progression of HCC cells [40]. UCK2 could induce cell cycle arrest and improve the immune response of HCC [41].

Subsequently, a nomogram was drawn to predict the survival probability of patients at 1-year, 3-year, and 5-year. The calibration plots and ROC curves in both training and test cohorts demonstrated that the nomogram could predict the survival of patients with HCC accurately. Meanwhile, Univariate and multivariate Cox regression analyses confirmed that the risk score of the gene signature was an independent prognostic factor. Previous studies have found that hypoxia could activate specific signaling pathways to reshape the immune microenvironment, leading to immunosuppression and immune evasion [42]. Thus, we further analyzed immune cell infiltration and immune function enrichment between high-risk and low-risk groups to investigate the correlation between the risk scores and immune infiltrates. According to the results, there was an upregulation of activated dendritic cells, macrophages, follicular helper T cells, and regulatory T cells in high-risk groups, whereas B cells, interdigitating cells, mast cells, NK cells, and T helper cells were downregulated. Immune function analysis indicated that the APC co-inhibition, checkpoint, and MHC-class-I were significantly enriched in the high-risk group. In contrast, the low-risk group exhibited a significant enrichment in cytolytic activity, Type-I-IFN-Response, and Type-II-IFN-Response. Meanwhile, the TIDE score assessed that patients in high-risk groups had decreased immune response, indicating a lower chance benefiting from immune therapy. The results from the waterfall diagram showed a significantly higher frequency of TP53 and ZFHX4 mutations in the high-risk group compared to the low-risk group. Notably, TP53 mutations have been associated with multiple cancers [43] and ZFHX4 has been recently found to involve in ovarian cancer [44], esophageal squamous cell carcinoma [45], breast cancer [46], and glioblastoma [47]. The heatmap of correlation analysis between risk score and hallmark pathways showed that high-risk score correlated more with glycolysis, which is consistent with previous results. Single-cell analysis revealed that hepatocytes/fibroblasts/plasma cells might play an important role in the interaction between signature genes and hypoxia/glycolysis/lactate metabolism, and there was a crosstalk between the above three cell types. We subsequently found that 8 risk genes (ADAMTS5, CDCA8, LDHA, YBX1, EIF5B, CBX2, RTN3 and GP6D) interacted with the ligand-receptors of hepatocytes/fibroblasts/plasma cells. The PPI network indicated that CDCA8, LDHA, YBX1 might be the hub genes of the regulation of hypoxia/glycolysis/lactate metabolism in HCC.

Based on the above results, the hypoxia-glycolysis-lactate-related gene signature we built can effectively divide HCC patients into two groups. The high-risk group has a poor prognosis, a low immune response, and a higher mutation frequency, suggesting that the treatment for the high-risk group may be unsatisfactory. Immune infiltration and immune function also differed in the high- and low-risk groups. The high-risk group interacted more with hypoxia/glycolysis/lactate metabolism and mainly with glycolysis, which is possibly due to the crosstalk between hepatocytes/fibroblasts/plasma cells. Therefore, to improve the survival of HCC patients, exploring drugs that target hypoxia/glycolysis/lactate metabolism, especially glycolytic enzymes, may be a promising strategy.

However, our study has certain limitations. Biases in sample selection and data collection methods should be addressed and we should analyze and evaluate the data more comprehensively in the future research, which can be conducted through machine learning [48, 49]. Further investigation to validate the findings of our bioinformatics analysis was required. Our future research is supposed to focus on the mechanisms of signature genes especially CDCA8, LDHA, YBX1 that involved in the progression of HCC. Exploring the impact of these identified genes on the tumor microenvironment, the interaction between these genes and cells/signal pathways of the microenvironment, and the molecular mechanisms in HCC progression will be necessary for our future research. Last but not least, basic research on whether CDCA8, LDHA, and YBX1 regulate HIFs, glycolytic enzymes, and lactate metabolism in HCC cells will be the future key points of our study.

## Conclusion

In conclusion, hypoxia/glycolysis/lactate metabolism correlate with the prognosis of HCC patients, and the above gene signature we bulit has promise in the prognosis estimation and precision treatment for HCC.

### Electronic supplementary material

Below is the link to the electronic supplementary material.

Supplementary Material 1

## Data Availability

The datasets presented in this study can be found in online repositories. These can be found in the Gene Expression Omnibus (GEO) database (https://www.ncbi.nlm.nih.gov/geo), and The Cancer Genome Atlas (TCGA) database (https://portal.gdc.cancer.gov).
